# Emergency Colectomy for Obstruction in Microsatellite Instability Colonic Cancer: A Complete Response Following Neoadjuvant Immunotherapy

**DOI:** 10.7759/cureus.89891

**Published:** 2025-08-12

**Authors:** Lamribah Mohamed, Lahoucine Alzaz, Douah Dounya, Mohammed Ouazni, Mehdi Soufi

**Affiliations:** 1 Surgery, Mohammed VI Hospital Agadir, Agadir, MAR; 2 General Surgery, University Hospital Center Souss Massa, Agadir, MAR; 3 General Surgery, Faculty of Medicine and Pharmacy, Ibn Zohr University, University Hospital Center Souss Massa, Agadir, MAR

**Keywords:** complete pathological response, emergency colectomy, immunotherapy, msi-h colorectal cancer, neoadjuvant therapy

## Abstract

A 40-year-old man with a recent deep vein thrombosis presented with chronic abdominal pain, rectal bleeding, night sweats, and significant weight loss. Imaging and colonoscopy revealed a stenosing transverse colon adenocarcinoma with loss of *MSH2* expression, confirming microsatellite instability-high (MSI-H) status. Molecular tests were negative for *KRAS*, *NRAS*, and *BRAF* mutations. The patient received four cycles of pembrolizumab as neoadjuvant therapy but required emergency subtotal colectomy for recurrent obstruction. Histopathology showed no residual tumor, negative lymph nodes, and pT0N0M0 staging, indicating a complete pathological response. This case illustrates the potential of short-course neoadjuvant immunotherapy to induce complete regression in MSI-H colorectal cancer and supports its safe integration into the surgical management of such tumors, even in emergency settings.

## Introduction

Microsatellite instability-high (MSI-H) colorectal cancer accounts for approximately 15% of localized colorectal cancer cases and is more prevalent in the proximal colon [1]. Around 20-25% of MSI-H tumors are associated with Lynch syndrome, the most common hereditary colorectal cancer syndrome [2]. MSI-H tumors result from deficient mismatch repair (dMMR) due to inactivation of *MLH1*, *MSH2*, *MSH6*, or *PMS2*, leading to the accumulation of insertion-deletion mutations at microsatellite regions [3]. This high mutational burden generates numerous tumor-specific neoantigens, making these tumors highly immunogenic and responsive to immune checkpoint inhibitors (ICIs) [4].

ICIs, such as pembrolizumab and nivolumab, target programmed cell death protein 1/programmed death ligand 1 or cytotoxic T-lymphocyte-associated protein 4 pathways, restoring antitumor T-cell activity [5]. Initially approved for metastatic MSI-H colorectal cancer, their role has expanded to earlier disease stages. The NICHE-2 trial demonstrated a pathological complete response (pCR) rate of 67% and a major pathological response rate of 95% after only two cycles of neoadjuvant nivolumab and ipilimumab in non-metastatic dMMR colon cancer [6]. Neoadjuvant therapy refers to treatment given before surgery to shrink tumors and improve resectability. pCR indicates the absence of viable tumor cells on histological examination post-treatment.

Here, we present a case of locally advanced MSI-H colon cancer treated with neoadjuvant pembrolizumab, complicated by bowel obstruction requiring emergency surgery, and achieving a complete pathological response.

## Case presentation

A 40-year-old man with no significant medical history, except for a recent episode of deep vein thrombosis of the lower limb, presented with chronic periumbilical abdominal pain, intermittent rectal bleeding, night sweats, and a weight loss of 16 kg over one year.

Initial laboratory tests revealed microcytic anemia (hemoglobin: 9.8 g/dL; reference: 13.5-17.5 g/dL), thrombocytosis (480 × 10⁹/L; reference: 150-400 × 10⁹/L), leukocytosis (13.2 × 10⁹/L; reference: 4.0-10.0 × 10⁹/L), and elevated C-reactive protein (85 mg/L; reference: <5 mg/L), indicating chronic blood loss and systemic inflammation (Table 1).

**Table 1:** Laboratory findings on admission.

Parameter	Patient’s value	Normal range
Hemoglobin	9.8 g/dL	13.5–17.5 g/dL (male)
Platelet count	480 × 10⁹/L	150–400 × 10⁹/L
White blood cell count	13.2 × 10⁹/L	4.0–10.0 × 10⁹/L
C-reactive protein	85 mg/L	<5 mg/L
Carcinoembryonic antigen	<1.7 ng/mL	<5 ng/mL
Cancer antigen 19-9	4 U/mL	<37 U/mL

**Figure 1:**
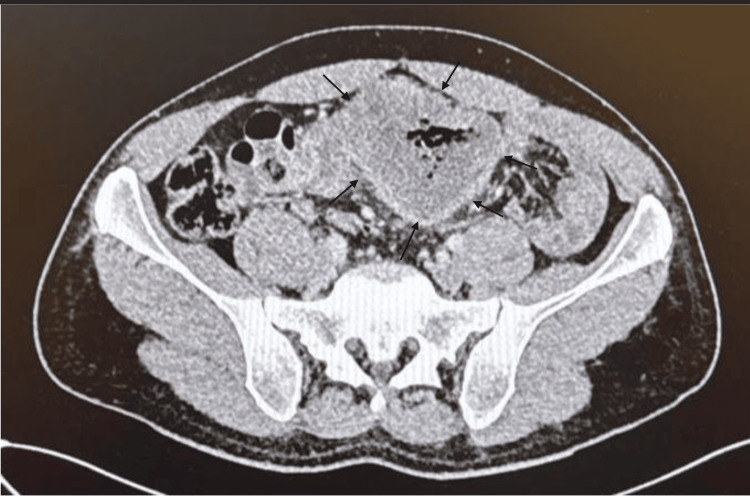
Circumferential and irregular thickening of the sigmoid colon over approximately 11 cm, with moderate mesenteric fat infiltration and perilesional lymph nodes.

An abdominopelvic CT scan demonstrated a circumferential and irregular thickening of the sigmoid colon extending over approximately 11 cm, associated with moderate mesenteric fat infiltration and perilesional lymphadenopathy (Figure 1).

Colonoscopy revealed a stenosing tumor located in the transverse colon. Histopathological examination identified a moderately to poorly differentiated adenocarcinoma with loss of *MSH2* expression, consistent with dMMR and confirming MSI-H status. Tumor markers were within normal limits, and molecular analysis showed no *KRAS*, *NRAS*, or *BRAF* mutations. Based on these findings, the diagnosis of MSI-H T4N+ colonic adenocarcinoma was established, strongly suggestive of Lynch syndrome.

Given the locally advanced nature of the tumor and the suspected involvement of adjacent structures, a neoadjuvant immunotherapy strategy was initiated. The patient received four cycles of pembrolizumab. Shortly thereafter, he was readmitted with signs of intestinal obstruction, prompting the need for emergency surgical intervention.

Although elective surgery had been initially scheduled for September 1st, it was brought forward to August 27th, due to a recurrence of obstructive symptoms. An emergency subtotal colectomy was performed via laparotomy, with a lateral-lateral ileosigmoid anastomosis. The postoperative course was uneventful, and the patient was discharged on September 5th.

**Figure 2:**
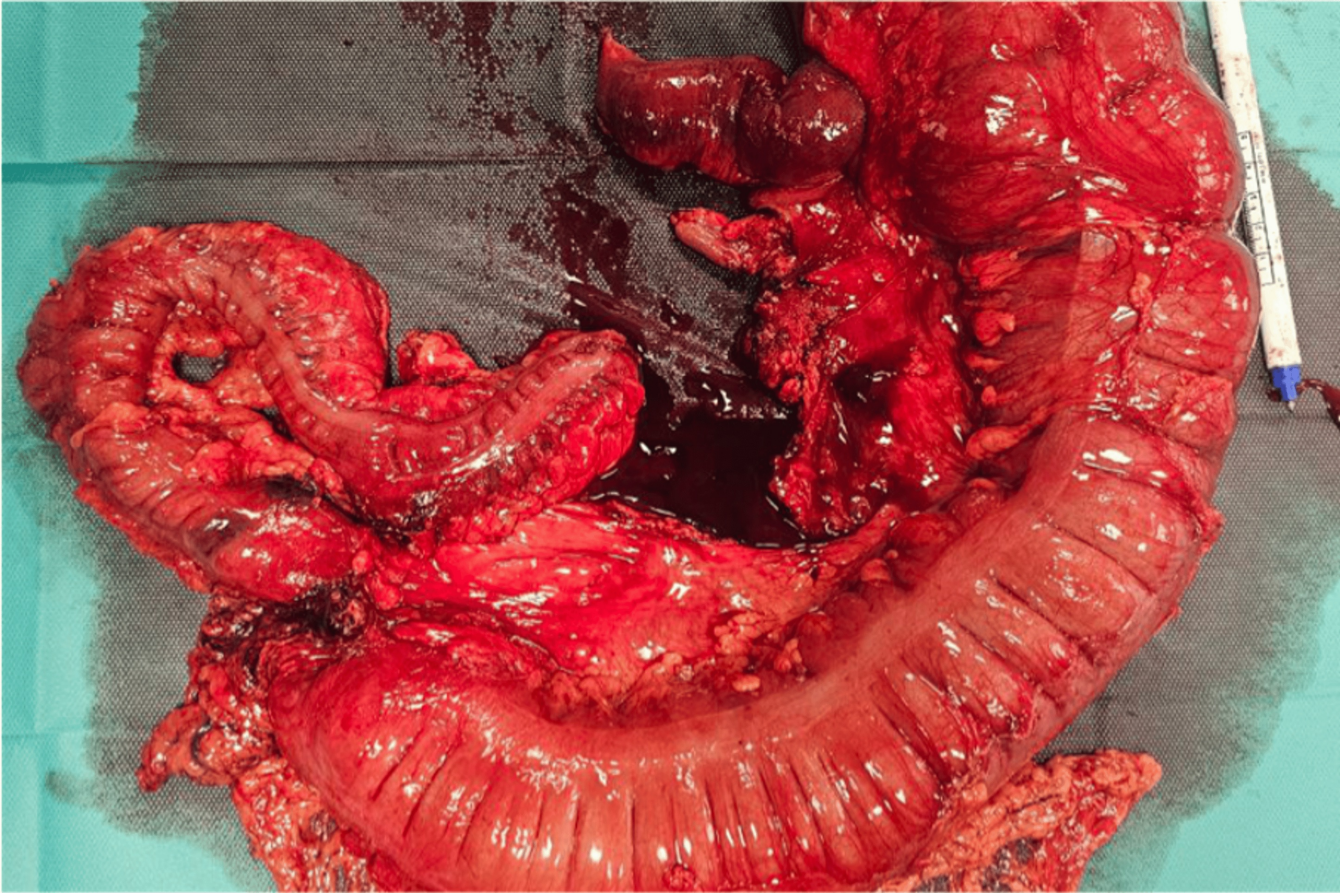
Resected colectomy specimen showing a fibrotic stenotic area with no visible tumor.

Macroscopic and histological examination of the resected specimen revealed no residual tumor cells, but rather a fibrotic scar at the tumor site. None of the 49 lymph nodes examined showed metastatic involvement, and both peritoneal cytology and omental tissue were negative for malignancy (Figure 2). These findings confirmed a complete pathological response to neoadjuvant immunotherapy, corresponding to a final staging of pT0N0M0.

## Discussion

This case underscores the remarkable efficacy of neoadjuvant immunotherapy in localized MSI-H colorectal cancer, even in the challenging context of an emergency surgical indication. MSI-H tumors, which account for approximately 15% of localized colorectal cancer cases, are characterized by dMMR resulting from mutations or epigenetic silencing of key DNA repair genes such as *MLH1*, *MSH2*, *MSH6*, or *PMS2* [1-3]. This defect leads to an accumulation of insertion-deletion mutations at microsatellite sequences, creating a high tumor mutational burden. The resulting abundance of tumor-specific neoantigens triggers strong immune recognition and makes these tumors particularly sensitive to ICIs [4].

In this patient, four cycles of pembrolizumab resulted in complete histological tumor regression (pT0N0M0), despite the treatment course being interrupted by an acute bowel obstruction. This observation aligns with data from pivotal trials, such as the NICHE-2 study, which reported pCR rates as high as 67% after only two cycles of neoadjuvant nivolumab plus ipilimumab in non-metastatic dMMR colon cancer [6]. Our case reinforces the concept that tumor clearance may occur rapidly, and prolonged preoperative exposure to ICIs is not always required to achieve a curative effect.

Another important aspect is the interpretation of post-treatment imaging. Radiological persistence of bowel wall thickening or mass-like lesions after neoadjuvant ICI therapy does not necessarily indicate residual viable tumor. As shown in a previous study, such findings may correspond to immune-mediated inflammation or fibrosis, leading to false-positive radiological impressions of disease persistence [7]. In our case, CT imaging before surgery continued to suggest a mass despite complete histological remission, illustrating the limitations of conventional radiology in the setting of ICI-treated dMMR colorectal cancer. This highlights the need for better imaging biomarkers to distinguish active disease from treatment-related changes.

Perioperative safety is another key consideration. Concerns have been raised that recent ICI exposure could potentially increase surgical complications due to immune-mediated tissue effects. However, available evidence suggests that surgery after ICI treatment is generally safe, with no significant increase in wound healing problems, anastomotic leaks, or infectious complications [8]. In our patient, emergency colectomy was performed without difficulty, and postoperative recovery was uneventful, supporting the feasibility of urgent surgery following neoadjuvant immunotherapy.

From a genetic standpoint, the loss of *MSH2* expression in a relatively young patient with no confirmed family history is highly suggestive of Lynch syndrome. This has significant implications for long-term surveillance and genetic counseling, both for the patient and for potentially affected relatives [2]. Early identification of such syndromes allows targeted screening and prevention strategies that can reduce morbidity and mortality from associated malignancies.

Finally, this case illustrates the importance of a multidisciplinary approach in the management of complex colorectal cancer cases. The integration of advanced molecular diagnostics, systemic immunotherapy, and timely surgical intervention, whether elective or emergent, maximizes the likelihood of achieving optimal oncologic outcomes. It also underscores the need for flexibility in surgical planning, as emergent situations may arise even during an effective systemic therapy.

In summary, neoadjuvant immunotherapy with ICIs represents a transformative advancement in the treatment of localized MSI-H colorectal cancer. Our case adds to the growing body of evidence supporting its use, demonstrates that complete pathological responses can be achieved after short treatment durations, and confirms that emergency surgery after ICI therapy can be both safe and effective.

In this case, surgery was dictated by recurrent bowel obstruction. However, it is notable that the emergency colectomy was feasible and uneventful, despite prior ICI exposure. This supports the safety of immunotherapy in the preoperative setting, even when the surgical intervention is unplanned.

Furthermore, the patient was relatively young and had no family history formally confirmed, but the loss of MSH2 protein strongly suggests an underlying Lynch syndrome. This adds an additional layer of importance to genetic counseling and tailored long-term surveillance for both the patient and potentially at-risk family members.

Overall, this case illustrates how a coordinated, multidisciplinary approach, integrating modern oncologic therapies with urgent surgical care, can lead to optimal outcomes in patients with immunogenic colorectal tumors.

## Conclusions

Neoadjuvant immunotherapy in localized MSI-H colorectal cancer can achieve complete pathological responses, even when surgery is performed urgently. This case supports incorporating ICIs into treatment algorithms for immunogenic tumors, highlights their perioperative safety, and underscores the importance of early molecular testing for optimal patient management.
